# H*fe* Gene Knock-Out in a Mouse Model of Hereditary Hemochromatosis Affects Bodily Iron Isotope Compositions

**DOI:** 10.3389/fmed.2021.711822

**Published:** 2021-10-15

**Authors:** Emmanuelle Albalat, Thibault Cavey, Patricia Leroyer, Martine Ropert, Vincent Balter, Olivier Loréal

**Affiliations:** ^1^CNRS UMR 5276, LGL-TPE, ENS de Lyon, Université de Lyon, Villeurbanne, France; ^2^INSERM, Univ Rennes, INRAe, UMR 1241, Plateforme AEM2, CHU Pontchaillou, Institut Nutrition Metabolisms et Cancer (NuMeCan), Rennes, France

**Keywords:** iron isotope, organs, mouse model, iron overloaded, hereditary hemochromatosis (HFE)

## Abstract

Hereditary hemochromatosis is a genetic iron overload disease related to a mutation within the HFE gene that controls the expression of hepcidin, the master regulator of systemic iron metabolism. The natural stable iron isotope composition in whole blood of control subjects is different from that of hemochromatosis patients and is sensitive to the amount of total iron removed by the phlebotomy treatment. The use of stable isotopes to unravel the pathological mechanisms of iron overload diseases is promising but hampered by the lack of data in organs involved in the iron metabolism. Here, we use H*fe*^−/−^ mice, a model of hereditary hemochromatosis, to study the impact of the knock-out on iron isotope compositions of erythrocytes, spleen and liver. Iron concentration increases in liver and red blood cells of H*fe*^−/−^ mice compared to controls. The iron stable isotope composition also increases in liver and erythrocytes, consistent with a preferential accumulation of iron heavy isotopes in H*fe*^−/−^ mice. In contrast, no difference in the iron concentration nor isotope composition is observed in spleen of H*fe*^−/−^ and control mice. Our results in mice suggest that the observed increase of whole blood isotope composition in hemochromatosis human patients does not originate from, but is aggravated by, bloodletting. The subsequent rapid increase of whole blood iron isotope composition of treated hemochromatosis patients is rather due to the release of hepatic heavy isotope-enriched iron than augmented iron dietary absorption. Further research is required to uncover the iron light isotope component that needs to balance the accumulation of hepatic iron heavy isotope, and to better understand the iron isotope fractionation associated to metabolism dysregulation during hereditary hemochromatosis.

## Introduction

Iron (Fe) homeostasis tuning is essential as Fe deficiency or excess in the body can lead to clinical severe consequences. Hereditary Hemochromatosis (HH) is a Fe overload disease, which is one of the most common genetic disease in Western countries (1, 2). The first clinical symptoms appear classically around 40 years old in men and 50 years in women. In absence of treatment, HH may favor liver cirrhosis, diabetes, heart failure, and reduced life expectancy (1). The treatment is based on Fe depletion that requires to perform phlebotomies, which must be done weekly in the initial phase and then bimonthly during the maintenance phase (3, 4). Promising chelating agents exist but are not recommended in HH due to potential harmful side effects (4, 5). HH is mainly related to a mutation of the HFE gene that plays a role in the control of hepcidin expression, the master regulator of systemic iron metabolism. Hepcidin, by inducing the degradation of the ferroportin protein, the only known Fe exporter from cells toward plasma, limits Fe release and thus controls the level of the saturation of transferrin, the Fe transport protein in plasma (6). An increase of transferrin saturation exposes to Fe overload, especially in the liver, pancreas and heart (6–10). Therefore, regulation of hepcidin expression plays a major role in the maintenance of Fe homeostasis since there is no, or very limited, physiological mechanisms for Fe excretion. During HH, transferrin saturation increases above 45%, leading to the appearance of abnormal biochemical forms of Fe in plasma: the Non-Transferrin Bound Iron (NTBI). The NTBI is not fully characterized. It is associated to low molecular compounds such as citrate or to proteins such as albumin (11, 12). During its association to transferrin, Fe must undergo an oxidation step that is reported to be enzyme-dependent. Importantly, NTBI that is easily incorporated by liver, heart and pancreas plays a major role in the development of Fe overload, especially in the liver (11–13).

The Human adult organism contains between 3 and 4 g of Fe. Iron metabolism is typified by an important recycling from erythrocytes, which contain about 70% of total Fe that is associated to hemoglobin, the oxygen transporter. Senescent red blood cells are taken up by macrophages and Fe is recycled during the erythrophagocytosis process that is the daily most important source of Fe for plasma. Importantly, despite the fact that there is no active mechanism for Fe excretion, there are few losses (1–2 mg per day) that cannot be avoided, such as cell desquamation, urine, and bleeding. Therefore, an equivalent amount of dietary Fe must be taken up daily by enterocytes. The main sources of Fe for plasma are thus the recycling of erythrocytes by macrophages during the erythrophagocytosis process, the dietary Fe absorption through the duodenum, and also in certain circumstances, the release of stored Fe from hepatocytes (14).

Iron is composed of 4 stable isotopes (^54^Fe, ^56^Fe, ^57^Fe and ^58^Fe, with respective relative abundances 5.8%, 91.8%, 2.1% and 0.3%) which relative proportions, i.e. isotope composition hereafter noted δ^56^Fe, may vary in health (15–19) and disease, including HH (20–22). The blood δ^56^Fe value of HH bloodletting treated patients is significantly higher compared to healthy age matched controls (20–22), and increases as a function of total Fe removed by phlebotomy (23). Two main mechanisms, the increase of Fe absorption efficiency and Fe release from Fe storage organs, which are not exclusive, have been proposed to explain these observations. First, phlebotomy stimulates intestinal Fe absorption (5) leading to a hypothesized reduced intensity of isotopic fractionation between diet and blood and therefore an increase of the blood δ^56^Fe value (24). Second, to maintain Fe balance and ensure erythrocytes production after phlebotomy, storage organs enriched in heavy isotopes, are solicited to release Fe to the plasma. This iron is used to meet the increased need for erythrocyte heme production (21, 23).

In this study, in order to unravel the specific Fe isotope fractionation in HH, we measure the Fe isotope composition in liver, spleen and erythrocytes in a genetically engineered mouse model of HH, the knock-out H*fe*^−/−^ mouse model, and in wild type Hfe^+/+^ (WT) controls. Plasma transferrin saturation, plasma Fe concentration and hepcidin expression are also measured.

## Methods

### Animal Handling

The study was approved by the ethical Rennes committee for animal experimentation. 12 months old male C57BL/6J mice were included in the study within two groups i.e., control H*fe*^+/+^ mice (*n* = 6) and H*fe*^−/−^ mice (*n* = 6). Knock-out H*fe*^−/−^ mice were generated by crossing heterozygous animals as previoustly reported (25). All mice were housed in animal facilities (ARCHE) of UMS Biosit in Rennes and fed a standard diet CRM-E (Special Diet Services) with a Fe concentration of about 140 μg/g. Mice were sacrificed at 12 months to ensure that iron overload was effective in the H*fe*^−/−^ group. After anesthesia in the morning between 9 and 12 am, a trans-diaphragmatic intracardiac puncture was done to collect blood in sodium heparin tubes suitable for trace element analysis. Liver and spleen samples were frozen in liquid nitrogen, and stored at -80°C.

### Sample Preparation

Plasma, erythrocytes, liver and spleen samples were handled with special care in order to avoid environmental contamination. After blood sample centrifugation, plasma was taken and frozen in polypropylene cryotubes at−80°C. Blood cell pellets, mainly erythrocytes containing very high amount of Fe in hemoglobin, were washed three times in NaCl 0.9%, centrifuged in NaCl 0.9% (3,000 g/min), and were then aliquoted and frozen in polypropylene cryotubes at -80°C.

Liver and spleen samples were desiccated for 15 h at 120°C in an oven and were weighed. Aliquots of about 10 μl were collected from frozen erythrocytes and about 2 to 5 mg were taken from dried liver and spleen samples. Erythrocytes, liver and spleen samples were mineralized in Teflon tubes filled with 2 ml of concentrated HNO_3_ (Fisher Chemical – Optima Grade). The Teflon tubes were placed in a MARS6 (CEM) microwave with a temperature maintained at 180°C during 2 h. This procedure was repeated twice by adding 1 ml of concentrated HNO_3_ to ensure a total mineralization of the samples. Dissolved samples were preserved at 4°C until elemental or isotopic analysis.

### Elemental and Isotopic Analysis

A small aliquot (5%) was taken for the determination of Fe concentration by inductively coupled plasma mass spectrometry (ICP-MS, X-Series II, Thermo Scientific) at the ÆM2 Platform from University of Rennes 1/Rennes Hospital, as previously reported (26).

The remaining solution was evaporated to dryness and devoted to Fe chemical purification prior to isotope analysis. Iron was isolated by ion-exchange chromatography as described in details in previous study (27). Briefly, the dried residue was taken up in HCl 6N and evaporated to dryness twice to guarantee total removal of remaining nitrate from the digestion procedure. Iron was then fixed on AG1-X8 anionic resin in HCl 6N media with traces of H_2_O_2_ in which the matrix elements pass through the column and are discarded. The elution of Fe was achieved by diluted HNO_3_ 0.5N and the total isolation procedure was repeated twice. Iron isotope compositions were measured using a Neptune Plus multi-collector inductively coupled plasma mass spectrometer (MC-ICPMS, Thermo Scientific) in medium mass resolution mode at ENS de Lyon. Iron solutions were diluted to 0.9 mg/L and doped with Ni at the same concentration to monitor and correct for instrumental mass fractionation. The instrument settings and analytical conditions are described in previous work (27). All the Fe isotope compositions are given in the delta annotation (expressed in ‰) and reported relative to the international isotopic standard NIST IRMM-014 with the following formula:


$$\begin{array}{l} \delta^{56/54}Fe_{IRMM14}=\left[\frac{\left(\frac{{\;}^{56}Fe}{{\;}^{54}Fe}\right)sample}{\left(\frac{{\;}^{56}Fe}{{\;}^{54}Fe}\right)std}-1\right] \end{array}$$

And will be hereafter abbreviated δ^56^Fe.

### Plasma Iron Concentration and Transferrin Saturation

Plasma Fe concentration and unsaturated iron-binding capacity (UIBC) were measured on Cobas 8,000 analyzer (Roche), by colorimetric method using the kits Iron Gen.2–750 tests (Roche) and Unsaturated Iron-Binding Capacity−100 tests (Roche), respectively. Plasma transferrin saturation (%) was then calculated as [Fe / (Fe + UIBC)] ^*^ 100.

### Expression of Hepatic Hepcidin

The expression level of hepcidin 1 mRNA transcripts was determined in the liver of WT and H*fe*^−/−^ mice by real time quantitative polymerase chain reaction (RT-PCR). Total liver RNAs were isolated using the Nucleospin 8 RNA (Macherey-Nagel). The mRNAs were reverse transcribed with the M-MLV reverse transcriptase (Promega). The following primers were used to amplify hepcidin 1 (forward: 5′-TTCCCAGTGTGGTATCTGTTGC-3′ and reverse: 5′-GGTCAGGATGTGGCTCTAG GC-3′), and TBP (TATA-binding protein) (forward: 5′-AAACTCTGACCACTGCACCG-3′ and reverse: 5′-GTGTGGCAGGAGTGATAGGG-3′) as references. Real-time quantitative PCR assays were performed using the qPCR MasterMix Plus for SYBR Green I (Eurogentec) and the system StepOne Plus (Real-Time PCR System–Applied Biosystems). All results were analyzed by StepOne Software v2.1 (Applied Biosystems). For each cDNA sample, the difference between the threshold cycle for hepcidin 1 amplification and the threshold cycle for TBP was calculated (ΔCt). This enables normalization of the amount of target to the TBP endogenous reference. Values of gene expression were expressed relatively to the control group's mean value (ΔCt-M). M corresponds to the ΔCt mean in the control group (WT mice). Results were expressed as [2^∧(−(ΔCt−M))^] ^*^ 100 in arbitrary unit (au).

## Results

**Figure 1:**
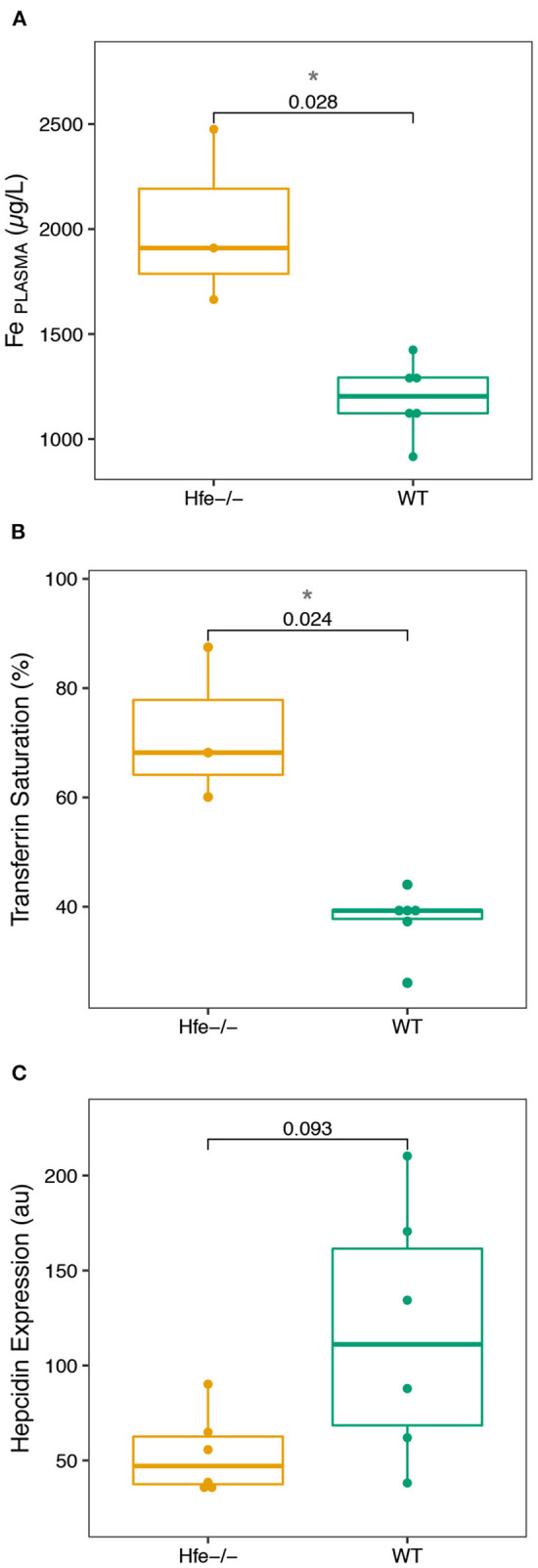
Boxplots of the biological parameters commonly investigated in hereditary hemochromatosis disease measured in the H*fe*^−/−^ mouse model and compared to the WT group. **(A)**, Fe plasma concentration. **(B)**, Transferrin Saturation. **(C)**, Hepcidin Expression. The *P*-values of Wilcoxon test and associated significance (***P < 0.001, **P < 0.01, *P < 0.05) are given.

All the results are given in Supplementary Tables 1, 2. We first assess plasma and liver biochemical parameters which are shown in Figure 1. As expected, a significant increase of the Fe concentration in plasma (Figure 1A), and transferrin saturation (Figure 1B) is found in H*fe*^−/−^ mice, compared to WT mice. Hepatic hepcidin mRNA level is lower in H*fe*^−/−^ than in WT mice, although the difference does not reach the significance threshold (*P-*value = 0.093; Figure 1C).

**Figure 2:**
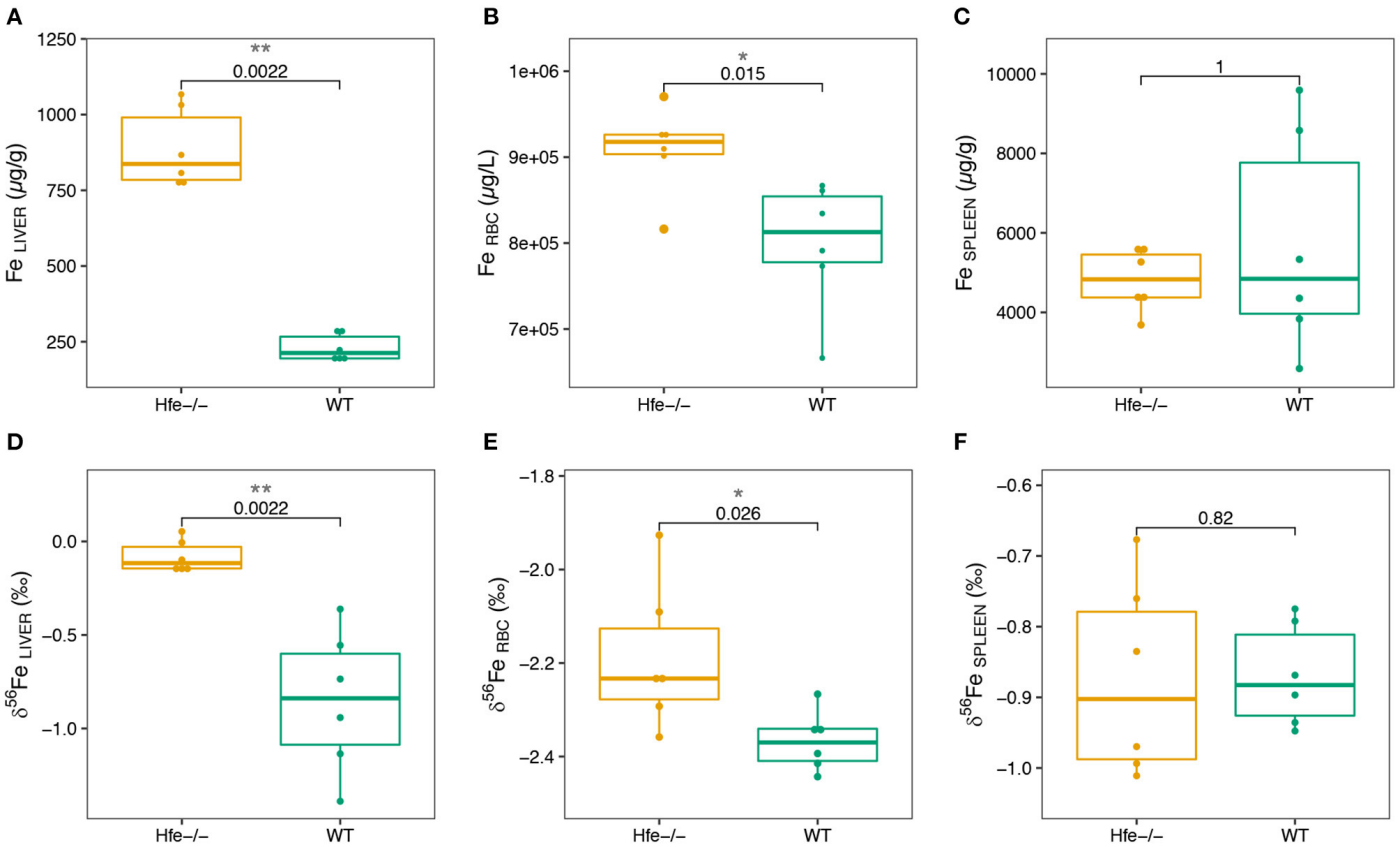
Boxplots of Fe concentration in organs of the H*fe*^−/−^ mouse model compared to the WT group **(A–C)** and of Fe isotope composition in organs of the H*fe*^−/−^ mouse model compared to the WT group **(D–F)**. RBC stands for erythrocytes. The *P*-values of Wilcoxon test and associated significance (***P < 0.001, **P < 0.01, *P < 0.05) are given.

We next explore Fe concentration and isotope composition in liver, erythrocytes and spleen. Results are presented in Figure 2; Supplementary Table 2. As expected, the hepatic Fe concentration is significantly increased in H*fe*^−/−^ compared to WT mice (Figure 2A). In erythrocytes, the Fe concentration is slightly but significantly increased in H*fe*^−/−^ compared to WT mice (Figure 2B). Iron concentration in spleen is not significantly different between the two groups (Figure 2C). The quality of Fe isotopic analysis was assessed by a three isotopes plot (Supplementary Figure 1). All the measured isotope ratios form a line indistinguishable within errors from theoretical predictions of both kinetic and thermodynamic isotope fractionation lines, implying no sizeable contribution of isobaric interferences on the isotopic measurements.

In liver and erythrocytes, the δ^56^Fe values are significantly more positive in H*fe*^−/−^ than in WT mice (Figures 2D,E; Supplementary Figure 2). In spleen, the Fe isotope composition and concentration are not different between the two groups (Figure 2F; Supplementary Figure 2). Liver and spleen are enriched in heavy Fe isotopes relative to RBC (Supplementary Figure 2), consistent with previous studies on WT mice (28), healthy minipigs (29) and human (15, 30).

## Discussion

The biological parameters that we have determined in the H*fe*^−/−^ mice compared to control animals (Figure 1) are consistent with those previously reported in H*fe*^−/−^ mice and humans during HH (4). The increase of serum Fe concentration and transferrin saturation, together with hepatic but not splenic Fe concentration increase, sign an hepcidin deficiency syndrome, confirmed by the absence of hepatic hepcidin mRNA level expression increase that is expected in condition of Fe overload (3, 4, 7).

**Figure 3:**
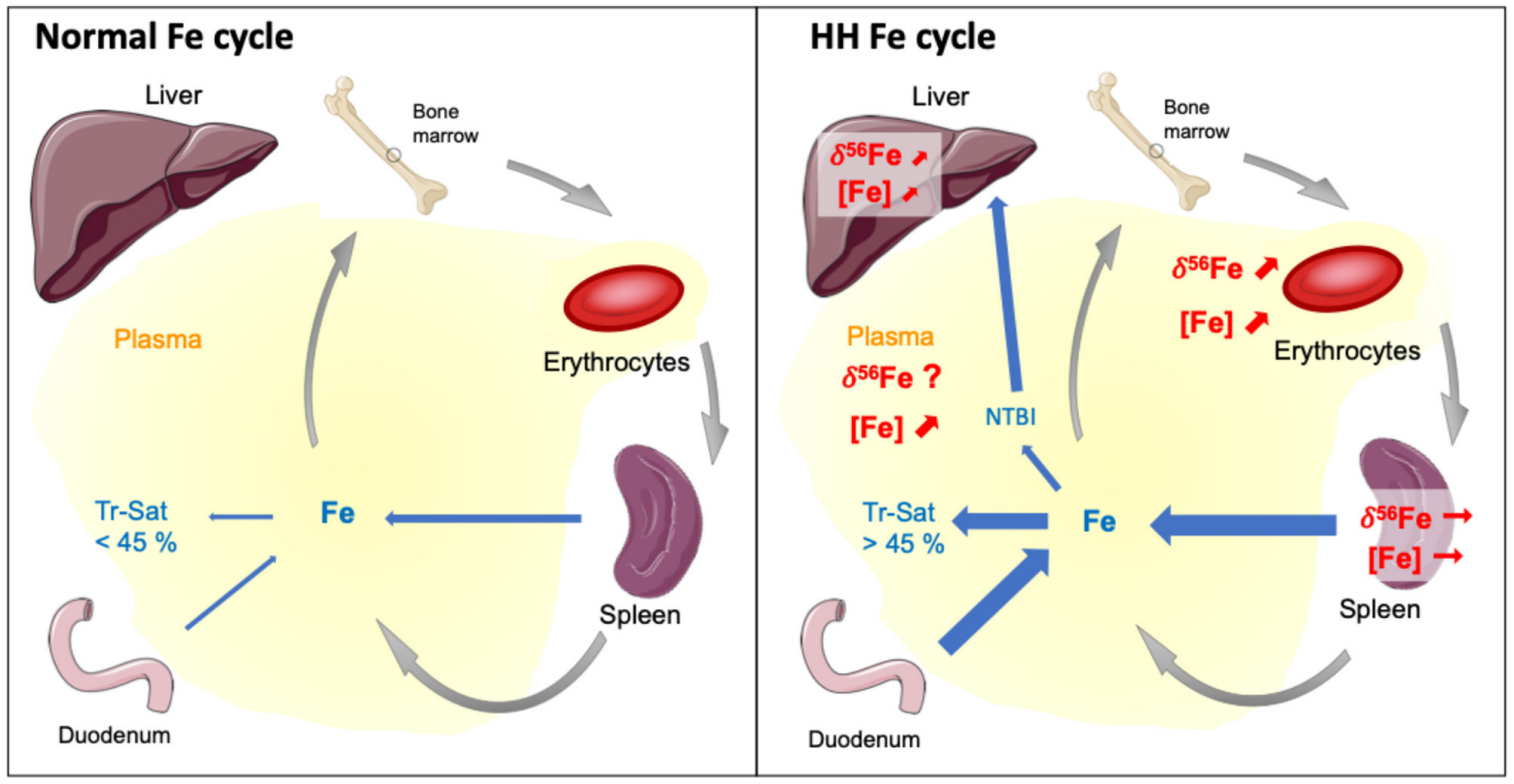
Schematic representation of the mechanisms leading to iron overloaded during H*fe*-related hemochromatosis according to general knowledge and conclusions of this study. Tr-Sat and NTBI stand for Transferrin saturation level and Non-Transferrin Bond Iron, respectively. In normal situation, the regulation of hepcidin expression allows control of Tr-Sat (<45%). Genetic hemochromatosis results in iron release in plasma from the dietary absorption through the duodenum and the recycling of erythrocytes by macrophages during the erythrophagocytosis process. The Tr-Sat increases above 45%, leading to the appearance of abnormal biochemical form of iron in plasma, the NTBI. The present study shows that Fe concentrations and isotope compositions of liver and erythrocytes, but not spleen, increase in a mouse model of HH compare to wild type. Our results suggest that the altered erythrocyte Fe isotope composition of HH patients originates from the disease and is aggravated by the cure. The heavy isotopes iron accumulation in liver could be related to the NTBI whose role should be confirmed by further studies.

Our study shows for the first time that the liver is enriched in Fe heavy isotope in H*fe*^−/−^ mice compared to control animals (Figure 2D). The existence of ^56^Fe-enriched hepatic stores in H*fe*^−/−^ mice might reflect preferential dietary absorption of heavy Fe isotopes and their presentation to the liver via the portal circulation. Without being exclusive, we propose that the accumulation of Fe heavy isotopes in liver could also be related to the NTBI known to appear in plasma when transferrin saturation increases during hemochromatosis. A schematic representation of the process implied in Fe overloaded during H*fe*-related hemochromatosis is given in Figure 3. Mechanisms leading to hepatic heavy isotope enrichment could indeed implicate (i) differential Fe isotopic composition between transferrin iron and NTBI chemical forms and/or, (ii) isotope fractionation during the intracellular process following the NTBI cell ingress, including associations with Fe chaperone molecules (31, 32) and ferritin-related oxidative steps (33–35). Interestingly, when Fe is in excess in liver, transferrin Fe uptake is decreased, the expression of transferrin receptor 1 being strongly downregulated. However, NTBI may continue to enter in hepatocytes through the ZIP14 protein that is not downregulated by cellular Fe excess. Thus, in the double knock-out ZIP14 and H*fe*^−/−^ mouse model there is no Fe accumulation in liver (36), which is consistent with the hypothesis of NTBI hepatic accumulation during HH (37). NTBI should be enriched in heavy Fe isotopes to account for the liver isotopic signature. Importantly, in HH patients, it has been reported a rapid increase of the blood δ^56^Fe value during phlebotomy that cannot be explained by an increase of dietary absorption only (23). Following the above evidence found in H*fe*^−/−^ mice, we can hypothesize that the rapid increase of blood δ^56^Fe value of HH patients during phlebotomy (23) could be linked to the release of ^56^Fe-enriched hepatic stores necessary for *de novo* erythropoiesis compensating blood subtraction. Indeed, Fe depletion therapy that aims to avoid deleterious consequences of Fe excess on organs is based on the fact that the removal of red blood cells induces erythropoiesis, a process requiring high amounts of iron that are mobilized from iron stores especially in the liver, to compensate the loss of erythrocytes to avoid hypoxia (7). Further studies aiming to analyze animals of different age and/or with iron overloads of other origins will help to better understand the mechanisms involved in the appearance of isotopic fractionation in the liver.

Our study also shows for the first time, to the best of our knowledge, a significant increase of the Fe concentration in RBC of H*fe*^−/−^ compared to WT littermates (Figure 2A). This finding is consistent with the slight elevated hemoglobin concentration and inflated volume of erythrocytes reported in HH (38, 39). This increase of Fe concentration in erythrocytes is associated with higher δ^56^Fe value (Figure 2E). In humans, the increase of the whole blood δ^56^Fe value, which is equivalent to that of erythrocytes (40, 41), has also been observed in HH patients relative to controls, but is significant only for those patients who were treated by phlebotomy (21). The present data, obtained in animals non-expressing the Hfe protein, shows that Fe metabolism dysregulation in HH leads to an increase of the blood δ^56^Fe value, which could be further aggravated by bloodletting (23).

**Figure 4:**
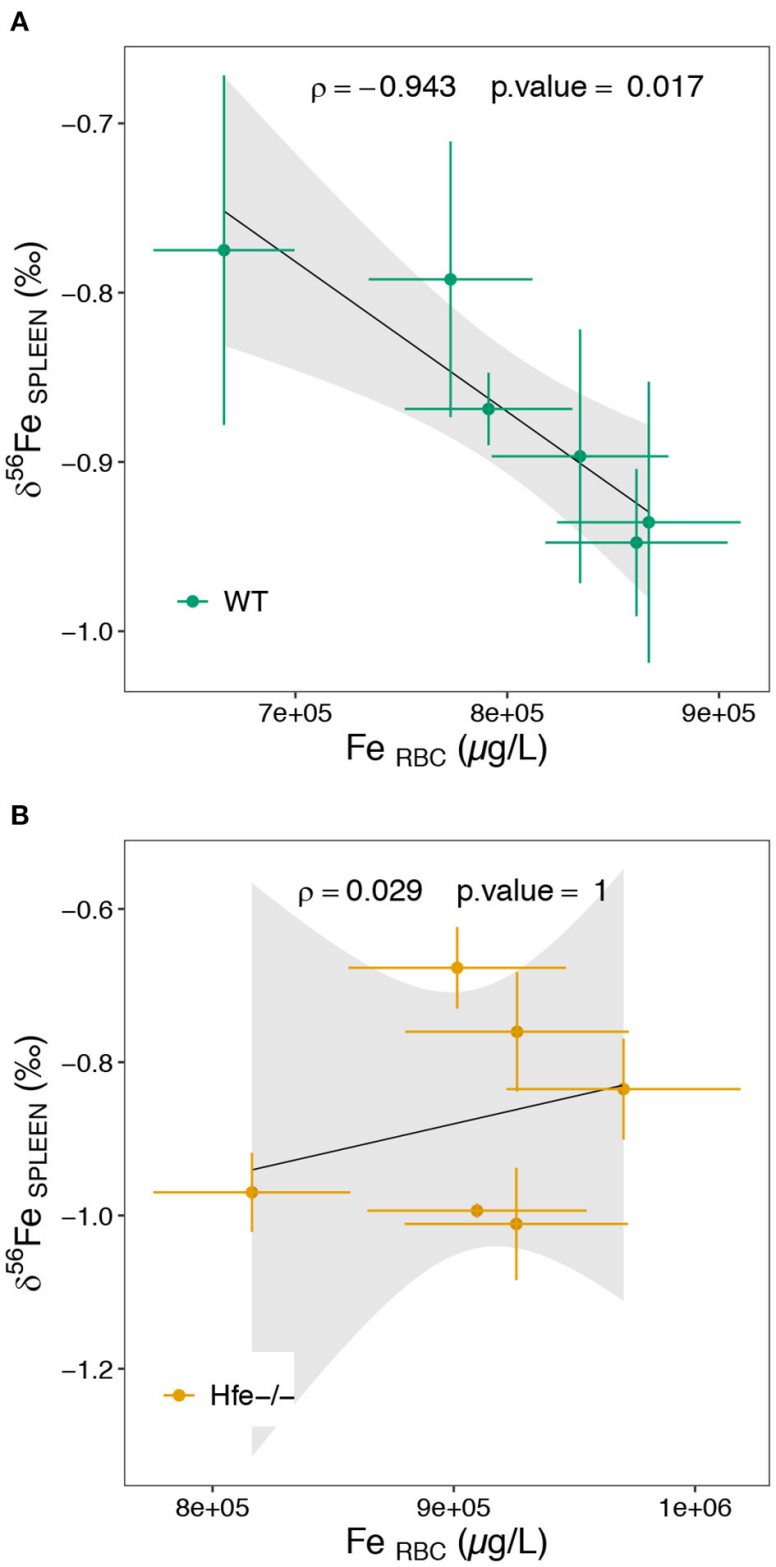
Scatterplot of the spleen Fe isotope composition as a function of the erythrocytes Fe concentration for the WT **(A)** and H*fe*-/- **(B)** mouse groups. RBC stands for erythrocytes. The ρ coefficient and *P*-values of the Spearman test with associated significance (***P < 0.001, **P < 0.01, *P < 0.05) are given.

In spleen, there is no difference in both Fe concentration and isotope composition between H*fe*^−/−^ and WT groups (Figures 2C,F). Splenic macrophages are part of the reticuloendothelial system, including specialized macrophage cells from spleen, liver and bone marrow, involved in Fe recycling from senescent erythrocytes. Splenic macrophages are thought to be the most active site of erythrocytes phagocytosis and Fe recycling (42, 43). Under normal physiological conditions, erythrocyte-recycled Fe is either released to the plasma by ferroportin, or stored in macrophages in ferritin if Fe is not needed in plasma. In HH, the hepcidin deficiency allows constant Fe release in plasma, despite Fe excess in plasma and liver. Also, in HH, the spleen is not overloaded such as it is observed here in the H*fe*^−/−^ mouse. In WT mice, the spleen δ^56^Fe value is correlated to erythrocytes Fe concentration (ρ = −0.94, ^*^P-value = 0.017; Figure 4A). Because erythrocytes have a very negative δ^56^Fe value (~−2.4‰), the amount of senescent erythrocytes in spleen will drive the spleen δ^56^Fe toward, negative values. This hypothetical inverse association does not exist in H*fe*^−/−^ mice (ρ = 0.03, *P*-value = 1; Figure 4B), and suggests that splenic reticuloendothelial cells, despite any obvious pathological expression in HH, have however a dysregulated Fe metabolism, with an apparent decoupling between erythropoiesis and erythrophagocytosis.

Further studies are required to fully depict the abnormal distribution of Fe in the HH disease. Notably, HH is characterized by a systemic accumulation Fe heavy isotope and further research is required to uncover the missing Fe light isotope component, that needs to balance the Fe isotopic budget. Unfortunately, we could not measure the plasma Fe isotope composition due to insufficient volume but this could be a challenge with dedicated and well designated further studies.

## Conclusion

The present study shows that the Fe isotope composition of liver and erythrocytes, but not spleen, are altered in a mouse model of HH. The Fe isotopic signature of liver can explain the origin of the increase of the whole blood δ^56^Fe value as a function of Fe removed by phlebotomy in HH patients, through the release of a Fe heavy isotope-enriched component. Our results suggest that the altered erythrocyte Fe isotope composition of HH patients originates from the disease and is aggravated by the cure. The role of abnormal biochemical forms of Fe, NTBI, that is avidly taken up by hepatocytes should be confirmed by further studies.

## Data Availability Statement

The original contributions presented in the study are included in the article/Supplementary Material, further inquiries can be directed to the corresponding author/s.

## Ethics Statement

The animal study was reviewed and approved by Comité Rennais d'éthique en matière d'expérimentation animale (CEEA-007).

## Author Contributions

OL and VB designed the study. PL performed animal experimentation. TC and MR contributed to ICP elemental analysis and biochemical assays. EA and TC performed isotopic analysis. EA performed the statistical analysis and wrote the first draft of the manuscript. All authors contributed to manuscript revision, read, and approved the submitted version.

## Conflict of Interest

The authors declare that the research was conducted in the absence of any commercial or financial relationships that could be construed as a potential conflict of interest.

## Publisher's Note

All claims expressed in this article are solely those of the authors and do not necessarily represent those of their affiliated organizations, or those of the publisher, the editors and the reviewers. Any product that may be evaluated in this article, or claim that may be made by its manufacturer, is not guaranteed or endorsed by the publisher.
