# Supplementary material for: Hfe Gene Knock-Out in a Mouse Model of Hereditary Hemochromatosis Affects Bodily Iron Isotope Compositions
Source: Front Med (Lausanne). 2021 Oct 15;8:711822. doi: 10.3389/fmed.2021.711822 (PMC8554230; doi:10.3389/fmed.2021.711822)
Supplement: Supplementary file 1 [file Data_Sheet_1.docx]

Supplementary Material

Fig S1: Mass fractionation in three-Isotope space for all the samples measured in this study. The linear regression is indistinguishable within errors from theoretical lines of mass-dependent isotope fractionation following exponential (slope=1.488) or inverse (slope=1.475) laws. This demonstrates the analytical validity of the data without the contribution of unresolved isobaric interferences.

Fig S2: Iron isotopic composition of RBC, spleen and liver of the two groups of mice.

The organs are enriched in heavy isotopes relative to RBC.

Table S1: Classical biological parameters considered in hereditary hemochromatosis disease associated with iron concentrations of liver, spleen and RBC.

Table S2: Iron isotopic compositions of liver, spleen and RBC of de 12 mice used in this study.
